# The importance of intraoral examination in the differential diagnosis of paracoccidioidomycosis

**DOI:** 10.1016/S1808-8694(15)30160-9

**Published:** 2015-10-18

**Authors:** Bruno Correia Jham, Anacelia Mendes Fernandes, Gabriela Versiani Duraes, Bruno Ramos Chrcanovic, Ana Cristina Rodrigues Antunes de Souza, Leandro Napier de Souza

**Affiliations:** a MSc; PhD in Oral and Experimental Pathology - University of Maryland, Baltimore; b MSc., Professor - Universidade Federal dos Vales do Jequitinhonha e Mucuri; c DDS; d DDS; e DDS. MSc in Materials Science - UFMG; f MSc. Professor - Centro Universitário Newton Paiva; g University of Maryland Dental School - Universidade Federal dos Vales do Jequitinhonha e Mucuri Centro Universitário Newton Paiva

**Keywords:** differential diagnosis, oral lesions, paracoccidioidomycosis, pneumonia, tuberculosis

## INTRODUCTION

Paracoccidioidomycosis (PCM) is a deep mycosis caused by the Paracoccidioides brasiliensis fungus. The primary disease affects the lungs, and can spread to other organs and tissues, creating secondary lesions.^1^

The most frequent systemic signs and symptoms are cough, lung secretion and weight loss.^2^ When present, the oral lesions are characterized by red and punctual micropapulae, settled amidst the whitish membrane of an ulcer.^3^

This paper reports two cases of PCM, initially diagnosed like other pulmonary diseases, where the definitive diagnosis was only possible after a histopathology test of the oral lesions.

## CASE PRESENTATION

### Case 1

**Figure 1:**
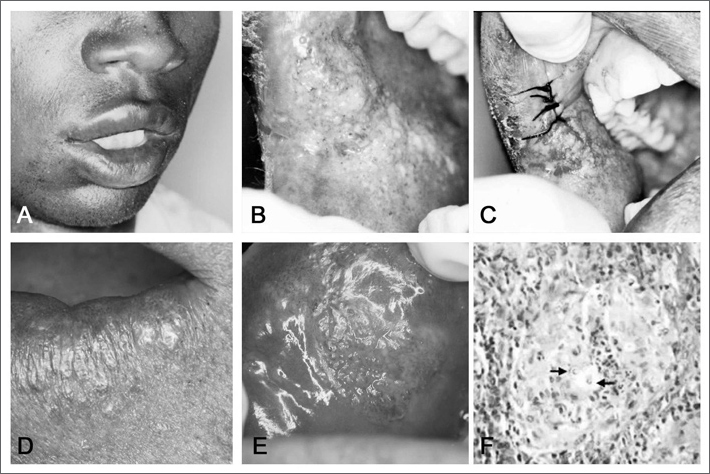
Clinical and histologic findings - (A) Extraoral aspect, showing macrocheilia; (B) moriform lesion in the oral mucosa; (C) suture after incisional biopsy; (D) Extensive crust lesion in the peri-oral region; (E) Ulceration in the upper lip mucosa with a moriform aspect; and (F) granulomatous chronic inflammation and the presence of the Paracoccidioides brasiliensis fungus (arrow).

Male, 24 years old, African-Brazilian, came to us complaining of a dental abscess. During the interview he reported a progressive weight loss in the past three years, cough, and he said he tested negative for neck lymph node tuberculosis. According to the patient, having the negative result of the lymph node biopsy, no other tests were carried out in order to find the cause of the symptoms, nor was any type of treatment installed. During the physical exam we observed macrocheilia and a moriform ulcerated lesion (Figs. 1A and 1B). After an incisional biopsy (Fig. 1C), the histopathology exam showed the presence of a granulomatous chronic inflammation, multinucleated gigantic cells and the Paracoccidioides brasiliensis fungus (Fig. 1F). The patient was then referred to the pneumologist, however the refused treatment.

### Case 2

Male, 37 years old, brown, the patient came to us complaining of a “sore mouth”. He reported one year before he had been admitted to the hospital for 23 days, with a suspected diagnosis of pneumonia. At the time he was treated with sulphametoxazole and trimethoprim by a pneumologist, however without improvement in his clinical condition. We observed an extensive crust lesion in his extra-oral exam (Fig. 1D). Moriform ulcers in his lips, oral mucosa and gums (Fig. 1E). The histopathology of his labial lesion showed a picture identical to the one described above in case 1 (Fig. 1F). The patient was referred back to his pneumologist, in order to reassess his treatment. According to the patient, he was submitted to a blood test that his doctor ordered and we did not have access to, before starting treatment with ketoconazole for three months and then sulphametoxazole + trimethoprim for six more months. The patient responded well to treatment and is now under control for four years.

## DISCUSSION

There are literature reports of misdiagnosed PCM in relation to other pulmonary diseases. Alves dos Santos et al.^4^ noticed that PCM frequently mimics pneumonia. Siletti et al.^5^ reported a case in which PCM was initially diagnosed as pneumonia caused by P.carinii.

Mayr et al.^6^ reported a case of PCM initially treated as if it were tuberculosis, even with bacterial culture and microscopic tests negative for the bacilli. Moreover, 10% of the patients with PCM also have tuberculosis.^1^ Anti-tuberculosis treatment may result in a partial improvement and make the clinician believe the patient was properly treated.

PCM causes oral lesions in about 50% of the patients, and the main differential diagnosis are squamous cell carcinoma and tuberculosis. Sarcoidosis and Wegener”s granulomatosis must also be considered.^3^

Swab cytology must be the first-choice procedure towards obtaining a diagnosis when oral lesions are present. In case of a positive test, treatment can be started immediately. Should that not be the case, the patient must be submitted to an incisional biopsy.^3^

Intravenous amphotericin B can be used for treatment, although it is very toxic. Ketoconazole or itraconazole also cause clinical cure, and they can be given orally.^3^

## FINAL REMARKS

The cases hereby reported show the importance of examining the patient”s oral cavity for the diagnosis of systemic diseases, including PCM. The fact that many medical conditions manifest themselves as oral lesions, as well as the ease of access, are strong justifications for the oral exam.

## References

[1] Borges-Walmsley MI, Chen D, Shu X, Walmsley AR (2002). The pathobiology of Paracoccidioides brasiliensis. Trends Microbiol.

[2] dos Santos JW, Severo LC, Porto Nda S, Moreira Jda S, da Silva LC, Camargo JJ (1999). Chronic pulmonary paracoccidioidomycosis in the state of Rio Grande do Sul, Brazil. Mycopathologia.

[3] Bicalho RN, Santo MF, de Aguiar MC, Santos VR (2001). Paracoccidioidomycosis: a retrospective study of 62 Brazilian patients. Oral Dis.

[4] Alves dos Santos JW, Torres A, Michel GT, de Figueiredo CW, Mileto JN, Foletto VG (2004). Non-infectious and unusual infectious mimics of community-acquired pneumonia. Respir Med.

[5] Silletti RP, Glezerov V, Schwartz IS (1996). Pulmonary paracoccidioidomycosis misdiagnosed as Pneumocystis pneumonia in an immunocompromised host. J Clin Microbiol.

[6] Mayr A, Kirchmair M, Rainer J, Rossi R, Kreczy A, Tintelnot K (2004). Chronic paracoccidioidomycosis in a female patient in Austria. Eur J Clin Microbiol Infect Dis.

